# Time-Enhancement Curve of Four-Dimensional Computed Tomography Predicts Aneurysm Enlargement with Type-II Endoleak after Endovascular Aneurysm Repair

**DOI:** 10.1055/s-0040-1702144

**Published:** 2020-07-31

**Authors:** Yunosuke Nishihara, Kota Mitsui, Shinya Azama, Daisuke Okamoto, Manabu Sato, Kozo Naito, Hitoshi Aibe

**Affiliations:** 1 Department of Radiology, Saga-Ken Medical Centre, Koseikan, Saga, Japan; 2 Department of Cardiovascular Surgery, Saga-Ken Medical Centre, Koseikan, Saga, Japan

**Keywords:** endovascular aneurysm repair, Type-II endoleak, computed tomography, time-enhancement curve

## Abstract

**Objective** We investigated the hemodynamic features of Type-II endoleaks after endovascular aneurysm repair (EVAR) using four-dimensional (4D) computed tomography (CT) to identify patients with aneurysm enlargement.

**Methods** During a 13-month period (January 2017–January 2018) at our institution, we performed 4D-CT examinations in 13 patients after EVAR because of suspected Type-II endoleaks. Three patients were excluded from the study because of other endoleaks or absence of detectable endoleaks. The ramaining 10 patients were divided into two groups: enlargement group ( *n* = 4), in which the aneurysm volume increased, and stable group ( *n* = 6), in which the aneurysm remained stable or shrank. A CT scanner and three-dimensional workstation were used. All images were obtained using a consistent protocol (22 phase scans using the test bolus tracking method). We analyzed the hemodynamics of the endoleak cavity (EC) relative to those of the aorta and evaluated the time-enhancement curves (TECs) using measurement protocols. The strengths of correlations between these factors in the two groups were analyzed statistically.

**Results** TECs in the enlargement group showed a more gradual curve, and the upslope, the gradient of TEC in the ascending phase and the upslope index were significantly more gradual than those in the stable group ( *p* = 0.0247, 0.0243). The EC washout and the EC washout index were also more gradual than in the stable group's ( *p* = 0.019, 0.019). The enhancement duration was longer in the former than in the latter (80%, *p* = 0.0195; 70%, *p* = 0.0159; 60%, *p* = 0.0159). The CT number in the equilibrium phase was larger in the enlargement group than in the stable group ( *p* = 0.019).

**Conclusion** The 4D-CT is useful for predicting aneurysm enlargement with Type-II endoleaks after EVAR.

## Introduction

The most frequent mechanism of failure after endovascular aneurysm repair (EVAR) is the occurrence of an endoleak. 1 2 Most endoleaks are Type II, defined as retrograde flow into the aneurysmal sac, most commonly from the patent inferior mesenteric artery (IMA), and/or lumbar arteries (LA).

The clinical significance of persistent Type-II endoleaks that last more than 6 months has not been defined. Although Types I and III endoleaks require immediate intervention, 1 3 the optimal treatment for persistent Type-II endoleaks is controversial, 4 even though aneurysm growth occurs in approximately 50% of such patients. 5 It is generally accepted that persistent Type-II endoleaks associated with aneurysm enlargement require aggressive management, because aneurysm enlargement is the most important predictor of aneurysm rupture. 6 It would therefore be beneficial to understand which imaging findings related to Type-II endoleaks are associated with the future need for intervention. To our knowledge, only a few small studies have examined a limited number of Type-II endoleak features and patient characteristics in an attempt to predict which Type-II endoleaks will progress, necessitating intervention, and which will not. 7 8 9 10

Conventional biphasic computed tomography (CT) angiography, usually consisting of arterial and venous phases, is the current standard method for pre- and postoperative imaging evaluations of abdominal aortic aneurysm. The recent introduction of four-dimensional computed tomography (4D-CT) has allowed the assessment of temporal enhancement patterns in the endograft, aneurysm sac, and adjacent aortic branches with a high temporal resolution. We designed the present study to reveal the hemodynamic features of Type-II endoleaks after EVAR using 4D-CT, to identify patients with aneurysm enlargement.

## Materials and Methods

### Patient Population

**Fig. 1:**
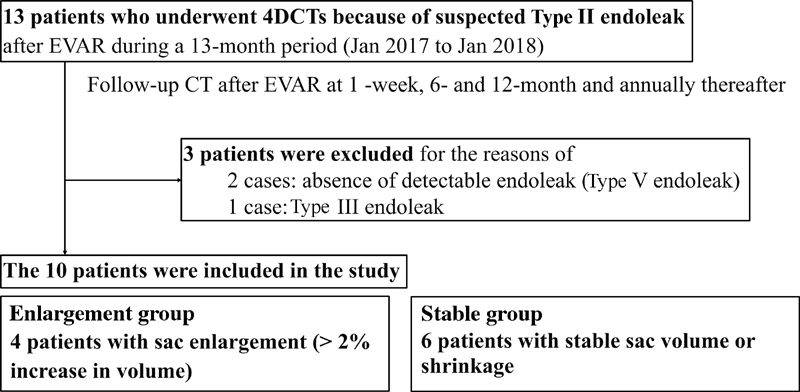
Flowchart of study profile. 4D, four-dimensional; CT, computed tomography; EVAR, endovascular aneurysm repair.

This was a case-control single-institutional study approved by our institutional review board. We obtained informed consent from all of the patients included. We performed 4D-CT examinations in 13 patients after EVAR with suspected Type-II endoleaks during a 13-month period (January 2017–January 2018) at Saga-Ken Medical Centre Koseikan ( Fig. 1 ). After EVAR, all of the patients underwent follow-up CT scans at the 1-week, 6- and 12-month follow-up points and annually thereafter in our institution. Three patients were excluded from this study because of Type-III endoleak ( *n* = 1) and absence of detectable endoleaks (Type-V endoleak; *n* = 2). The ramaining 10 patients were divided into two groups: enlargement group and stable group. Four patients were in the enlargement group where the aneurysm volume increased by >2%. In all these cases, the enlargement was confirmed at the next follow-up CT examinations. Six patients were in the stable group where the aneurysm volume remained the same or was reduced at the next follow-up CT examinations. This group included two cases in which endoleaks had disappeared by the time of the next examination.

### Image Acquisition

All CT studies were performed with a 320-row CT scanner (Aquilion ONE; Canon Medical Systems, Tochigi, Japan). Scanning parameters were kept as consistent as possible. The scanning parameters for 4D-CT were: acquisition, 22 phases; scan mode, volume; tube voltage, 100 kVp; tube current, 100 mA; rotation time, 0.5 second/rotation; collimation, 0.5 mm × 320; and CTDI _vol_ , 70.1 mGy. The radiation dose is almost the same as that of conventional follow-up CT examination consists of three times scanning (precontrast image and two phase contrast images). We injected contrast medium using the test bolus tracking (TBT) method. 11 Iopamidol was injected via the right antecubital vein at 2.5 to 4.5 mL/s (fractional dose: 24 mgI/kg/s) for 3 seconds, immediately followed by a saline flush injected for 5 seconds as a test bolus injection. After an interval of 15 seconds, the main bolus composed of contrast medium for 10 seconds followed by a saline flush for 5 seconds was injected. The TBT method made it possible to start scanning just before the main bolus arrived at the aorta. Equilibrium phase images were also acquired 90 seconds after the arrival time at the aorta. Acquired images were reconstructed and archived as 0.5-mm-thick sections with no overlap.

### Image Interpretation

**Fig. 2:**
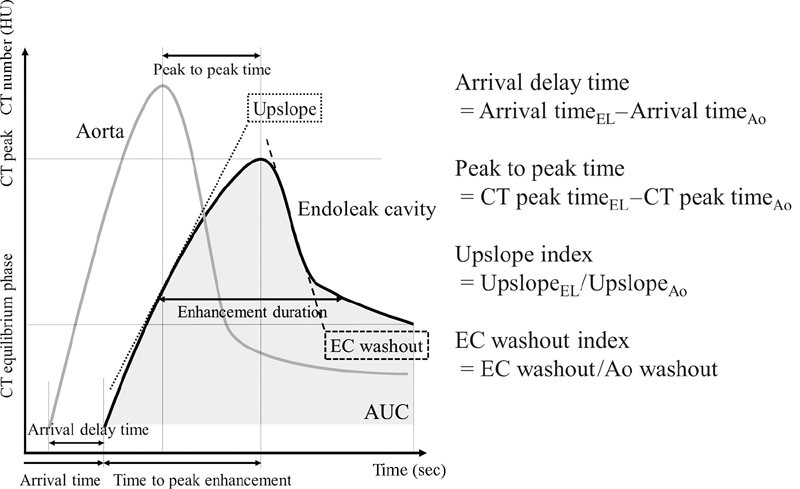
Measurement protocol of time-enhancement curve. AUC, area under curve; CT, computed tomography; EC, endoleak cavity.

All CT examinations in 10 patients with Type-II endoleaks were retrospectively measured in greater detail to evaluate the TEC of the endoleak cavity (EC) and that of the aorta. Measurements were performed by one observer (K.M. with 10 years' experience analyzing vascular CT images). Measurements of the TEC and aneurysm volumetry were performed using commercial image postprocessing software (Ziostation2 ver. 2.4.2.3, Ziosoft, Tokyo). The TEC of the aorta was measured by positioning a region of interest in the aorta at the level of the ostial superior mesenteric artery. Measurements of the TEC were performed by positioning a region of interest within the EC at a representative section away from feeding/draining arteries to avoid any hemodynamic influence of side-branch vessels. We evaluated the TEC using the measurement protocol shown in Fig. 2 . We also recorded arrival time; arrival delay time, the difference between the arrival time at the EC and at the aorta; time to peak enhancement, the difference between the CT peak time and the arrival time; maximal CT number, CT peak; and the CT number in equilibrium phase, 90 seconds after the arrival time at the aorta. We measured the gradient of the curve in the ascending phase as the upslope, and the gradient of the curve in descending phase as the EC washout. Peak-to-peak time was defined as the difference between the CT peak time at the EC and the aorta. The duration of contrast enhancement achieved within 80% (70 and 60%) of the peak was defined as 80% (70 and 60%) enhancement duration. We also measured the area under the curve (AUC) and the area under the TEC. We defined the upslope index and EC washout index to calibrate circulatory conditions. The upslope index was defined as the upslope of the EC divided by the upslope of the aorta. The EC washout index was defined as the EC washout divided by that of the aorta.

### Statistical Analysis

We performed univariate analyses for comparisons of the enlargement group and the stable group. Continuous variables were expressed as medians (interquartile range [IQR]); these were analyzed with the Mann–Whitney *U-* test for unpaired comparisons ( Table 1 ). All statistical analyses were performed with EZR (Saitama Medical Center, Jichi Medical University, Saitama, Japan), which is a graphical user interface for R (The R Foundation for Statistical Computing, Vienna, Austria). 12 More precisely, EZR is a modified version of R commander (ver. 1.33) designed to add statistical functions frequently used in biostatistics. Findings were considered significant when the *p* -value was <0.05.

**Table 1:** Time-enhancement curve characteristics comparisons of enlargement and stable groups

Parameter	Enlargement group *n* = 4	Stable group *n* = 6	*p* -Value
Arrival time (s)	15 (13–22)	9 (7–10)	0.106
Arrival delay time (s)	13 (12–16)	7 (6–10)	0.133
Time to peak enhancement (s)	17 (14–19)	12 (11–14)	0.234
CT peak (HU)	192 (130–262)	265 (227–319)	0.352
Upslope (HU/s)	7 (7–8)	24 (17–30)	**0.0247**
Upslope index	0.21 (0.18–0.26)	0.72 (0.49–1.04)	**0.0243**
EC washout (HU/s)	1.96 (1.31–3.38)	16.6 (14.6–21.2)	**0.019**
EC washout index	0.07 (0.05–0.12)	0.70 (0.57–0.82)	**0.019**
Peak to peak time (s)	17 (16–19)	5 (4–6)	0.108
80% enhancement duration (s)	14.8 (13.7–16.1)	7.4 (6.1–7.4)	**0.0195**
70% enhancement duration (s)	20.7 (18.9–22.2)	8.4 (8.3–8.6)	**0.0159**
60% enhancement duration (s)	29.9 (27.3–33.0)	10.6 (10.1–11.7)	**0.0159**
AUC (HU × s)	11,909 (11,642–12,117)	12,815 (9,766–15,661)	1
CT equilibrium phase (HU)	90 (75–103)	27 (22–39)	**0.019**

Abbreviations: AUC, area under curve; CT, computed tomography; EC, endoleak cavity; TEC, time-enhancement curve.

Note: all data are medians. Numbers in parentheses are the interquartile range (lower quartile[Q1]–upper quartile[Q3]).

## Results

### Time-Enhancement Curve Characteristics

There were many significant between-group differences in certain aspects of the TEC characteristics ( Table 1 ). There were no significant differences between the two groups in the arrival time, arrival delay time, time to peak enhancement, and CT peak. The upslope was significantly more gradual in the enlargement group than in the stable group: 7 HU/s, IQR, 7 to 8 versus 24 HU/s, IQR, 17 to 30 HU/s ( *p* = 0.0247). The upslope index was significantly smaller in the enlargement group than in the stable group: 0.21, IQR, 0.18 to 0.26 versus 0.72, IQR, 0.49 to 1.04 ( *p* = 0.0243). EC washout was also significantly more gradual in the enlargement group than in the stable group: 1.96 HU/s, IQR, 1.31 to 3.38 versus 16.6 HU/s, IQR, 14.6 to 21.2 HU/s ( *p* = 0.019). The EC washout index was also significantly smaller in the enlargement group than in the stable group: 0.07, IQR, 0.05 to 0.12 versus 0.70, IQR, 0.57 to 0.82 ( *p* = 0.019). There were no significant differences between the two groups in the peak to peak time. The enhancement duration time was significantly longer in the enlargement group than in the stable group at each percentage: 80%; 14.8 seconds, IQR, 13.7 to 16.1 versus 7.4 seconds; IQR, 6.1 to 7.4 seconds ( *p* = 0.0195); 70%; 20.7 seconds, IQR, 18.9 to 22.2 versus 8.4 seconds; IQR, 8.3 to 8.6 seconds ( *p* = 0.0159); 60%; 29.9 seconds, IQR, 27.3 to 33.0 versus 10.6 seconds; IQR, 10.1 to 11.7 seconds ( *p* = 0.0159). There were no significant differences in AUC between the two groups. The CT equilibrium phase was significantly higher in the enlargement group than in the stable group: 90 HU, IQR, 75 to 103 versus 27 HU, IQR, 22 to 39 HU ( *p* = 0.019).

## Discussion

The need for vigilant surveillance with optimized CT angiography protocols and image postprocessing methods is strongly supported by evidence of aneurysm rupture in patients with persistent Type-II endoleaks. 13 The most important parameter for indicating nonrupture in a patient with Type-II endoleak is a stable aneurysm sac volume on CT angiography images, and previous work has shown no significant differences in clinical characteristics (e.g., sex, smoking history, hypertension, need for anticoagulation, aneurysm diameter, and type of endograft used) between patients with enlarging aneurysms and those with either shrinking or stable aneurysms. 14 Other conventional CT angiography studies have revealed several indicators of aneurysm enlargement. Demehri et al 15 showed that the EC volume in patients with aneurysm enlargement was significantly larger than that in patients with stable or shrinking aneurysms. By analyzing delayed acquisition CT images, Timaran et al 8 demonstrated that the maximum diameter of an EC can be used to accurately indicate sac enlargement. Previous studies 8 16 have also showed that the median number of feeding and/or draining arteries was greater in patients with enlarged aneurysms than in patients with stable or reduced aneurysms. Müller-Wille et al 16 categorized Type-II endoleaks into more detailed subtypes, and they stated that the strongest indicators for aneurysm sac enlargement were complex IMA-LA Type-II endoleaks and the diameter of the largest feeding and/or draining artery.

**Fig. 3:**
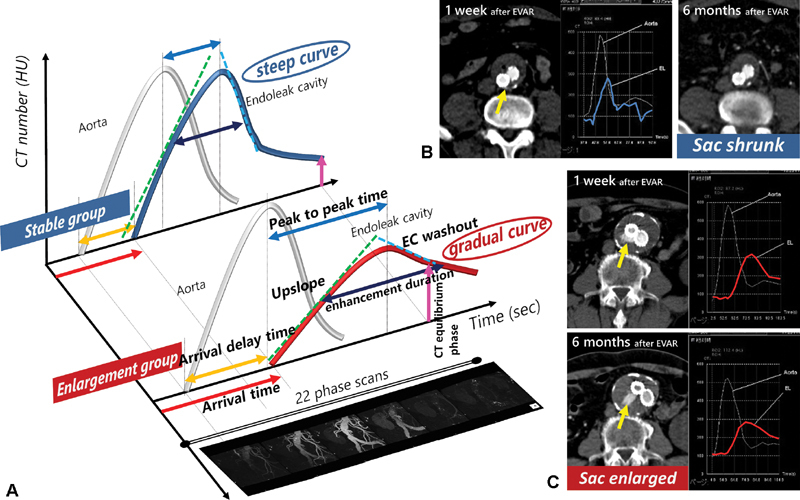
( **A** ) Schema of the time-enhancement curve (TEC) of a Type II endoleak. ( **B** and **C** ) Two actual cases. ( **B** ) A 71-year-old male underwent endovascular aneurysm repair (EVAR), and four-dimensional computed tomography (4D-CT) after 1 week revealed a tiny Type-II endoleak (yellow arrow). The TEC of the endoleak was steep (blue curve). Six months after EVAR, the Type-II endoleak had disappeared and the aneurysm sac had shrunk (30–23 cc). ( **C** ) An 85-year-old male underwent EVAR and 4D-CT after 1 week revealed a Type-II endoleak (yellow arrow). The TEC of the endoleak was gradual (red curve). Six months after EVAR, a persistent Type-II endoleak was found and the aneurysm sac was enlarged (69–79 cc). The TEC pattern was similar to that at 1 week after EVAR.

We analyzed the TEC of the EC and the aorta in 4D-CT images to evaluate the hemodynamic characteristics. We found that patients with sac enlargement had longer enhancement duration, a more gradual upslope and gradual EC washout and a higher CT equilibrium phase ( Fig. 3 ). That is, the TEC in patients with sac enlargement became more gradual. Bargellini et al 7 showed that a longer washout time (>520 seconds) was the only independent predictor of aneurysm sac enlargement in patients with persistent Type-II endoleaks identified by contrast-enhanced ultrasound scanning. Demehri et al 15 also reported four CT examinations in which Type-II endoleaks were observed and measured only in the delayed phase and demonstrated aneurysm enlargement. Their arguments are consistent with the results of our TEC analyses. That is, the TEC of the EC measured only in the delayed phase or with a longer washout time lay on a gradual curve with a gradual upslope and a gradual EC washout, and larger CT number at the equilibrium phase correlated with subsequent aneurysm enlargement. Dias et al 9 measured the intra-aneurysm sac pressure directly using tip-pressure sensors through direct translumbar puncture. In that study, Type-II endoleaks in shrinking aneurysms had a lower intrasac pressure than expanding or stable aneurysms. We presume that in patients with aneurysm shrinkage, a lower intrasac pressure induces a shorter arrival delay time and a shorter peak to peak time, which cause a steep TEC in 4D-CT. However, the arrival delay time and the peak to peak time in the stable group tended to be short overall than those in the enlargement group, but we could not prove statistical difference. We believe that a large cohort study will demonstrate more certain correlations.

Lehmkuhl et al 17 revealed that the peak enhancement of an endoleak is different than that of the aorta and that endoleaks may not be adequately evaluated using conventional biphasic CT protocols, suggesting that 4D-CT increases the endoleak detection rate. We consider that 4D-CT and TEC analyses can differentiate other types of endoleaks, detect small endoleaks, and furthermore can predict sac enlargement. Our search of the relevant literature did not identify any report suggesting the importance of the TEC for 4D-CT as an indicator of aneurysm sac enlargement.

### Limitations

Several limitations of our study should be considered. First, all of the patients in this study were diagnosed based on 4D-CT images; they did not undergo conventional angiography for evaluation of endoleaks which some researchers consider essential for proper classification of all endoleaks. 2 4 Second, we did not validate the accuracy of the volumetry using a phantom model of endoleaks. One observer obtained all of the measurements, and we were unable to determine the interobserver measurement variability. Finally, our patient series was somewhat small, thus preventing us from exploring other clinical applications of TEC analyses.

## Conclusion

The results of the present study demonstrated that 4D-CT is useful for predicting aneurysm enlargement with Type-II endoleaks after EVAR. TEC characteristics should be considered when interpreting follow-up CT examinations of patients with Type-II endoleaks, because this will provide an important guide for monitoring and determining the need for additional interventions in these patients.
